# Treatment and Prevention of Bone Metastases from Breast Cancer: A Comprehensive Review of Evidence for Clinical Practice

**DOI:** 10.3390/jcm3010001

**Published:** 2014-01-09

**Authors:** Bob T. Li, Matthew H. Wong, Nick Pavlakis

**Affiliations:** 1 Department of Medical Oncology, Royal North Shore Hospital, St. Leonards NSW 2065, Sydney, Australia; E-Mail: bob.li@med.usyd.edu.au; 2 Sydney Medical School, University of Sydney, Camperdown NSW 2050, Sydney, Australia; E-Mail: matthewhwong@uni.sydney.edu.au; 3 Department of Medical Oncology, Gosford Hospital, Gosford NSW 2250, Australia

**Keywords:** breast cancer, bone metastases, bisphosphonates, denosumab, skeletal-related events, adjuvant, randomized controlled trials, meta-analysis

## Abstract

Bone is the most common site of metastasis from breast cancer. Bone metastases from breast cancer are associated with skeletal-related events (SREs) including pathological fractures, spinal cord compression, surgery and radiotherapy to bone, as well as bone pain and hypercalcemia, leading to impaired mobility and reduced quality of life. Greater understanding of the pathophysiology of bone metastases has led to the discovery and clinical utility of bone-targeted agents such as bisphosphonates and the receptor activator of nuclear factor kappa-B ligand (RANK-L) antibody, denosumab. Both are now a routine part of the treatment of breast cancer bone metastases to reduce SREs. With regards to prevention, there is no evidence that oral bisphosphonates can prevent bone metastases in advanced breast cancer without skeletal involvement. Several phase III clinical trials have evaluated bisphosphonates as adjuvant therapy in early breast cancer to prevent bone metastases. The current published data do not support the routine use of bisphosphonates in unselected patients with early breast cancer for metastasis prevention. However, significant benefit of adjuvant bisphosphonates has been consistently observed in the postmenopausal or ovarian suppression subgroup across multiple clinical trials, which raises the hypothesis that its greatest anti-tumor effect is in a low estrogen microenvironment. An individual patient data meta-analysis will be required to confirm survival benefit in this setting. This review summarizes the key evidence for current clinical practice and future directions.

## 1. Introduction

Breast cancer is the most common malignancy and the leading cause of cancer death among women worldwide [1]. The incidence is highest in North America, Australia, New Zealand, western and northern Europe, where as many as one in eight women develop breast cancer [2,3]. Bone is the most common site of breast cancer metastasis, with over 70% of patients who died from breast cancer found to have bone metastases on postmortem examination [4]. In women with early breast cancer, risk factors for developing bone metastases include the presence of significant nodal disease with greater than four involved axillary lymph nodes at initial diagnosis, primary tumor size greater than 2 cm, estrogen receptor positive progesterone receptor negative tumor and younger age [5,6]. The presence of significant nodal disease has the highest cumulative incidence of bone metastases: 15% at 2 years and 41% at 10 years [5]. The median survival for patients with breast cancer and bone metastases is approximately 2 years [7,8]. Patients with bone-only metastases have a significantly better outcome than those with visceral metastases [8] and highly selected series have reported an average survival of 72 months [9]. However, bone metastases from breast cancer are associated with significant morbidity including immobility and the development of SREs, which are defined as the development of pathological fractures, spinal cord compression, the need for surgery and radiotherapy to bone. When bone pain and hypercalcemia are included as SREs as in older definitions, SREs occur in over 50% of patients with breast cancer bone metastases [10]. Given the frequency of breast cancer bone metastases, its negative impact on quality of life, the relatively longer survival of these patients and the burden of SREs to society, much research has focused on the pathophysiology, treatment and prevention of bone metastases from breast cancer over the last two decades. This paper discusses key research findings and summarizes the data from randomized controlled trials for evidence-based clinical practice and future directions. 

## 2. Pathophysiology of Bone Metastases

Normal bone formation is a coordinated dynamic process of active bone production by osteoblasts and bone remodeling and resorption by osteoclasts. This fine balance is mediated by a variety of local and systemic factors such as transforming growth factor-beta (TGF-β), insulin growth factor (IGF), bone morphogenic protein, platelet-derived growth factor (PDGF), prostaglandin and parathyroid hormone, as well as RANK-L, a key factor for osteoclast production [11]. Bone metastases disrupt this complex interplay through an organized and multistep process involving tumor intravasation, cell survival in the circulatory system, extravasation into surrounding tissue, initiation and maintenance of growth, vascularization and angiogenesis [12]. Key gene expression signatures identified in this process include C-X-C chemokine receptor type 4 (CXCR4), fibroblast growth factor 5, connective tissue-derived growth factor, interleukin-11, matrix metalloproteinase (MMP)-1, follistatin, A disintegrin and metalloproteinase with thrombospondin motifs 1 (ADAMTS1) and proteoglycan-1, all of which are overexpressed by at least four-fold when compared with the same cell lines that have not metastasized to bone [13].

The “seed and soil” hypothesis was first proposed in 1889 by Stephen Paget, who suggested that the “seeds (tumor) can only live and grow if they fall on congenial soil (optimal bone microenvironment)” [14]. This concept was further expanded in the “metastatic niche” model by Psaila and Lyden, who explained the relationship between the disseminating seed (tumor) and metastatic soil (bone) through a continual supply of growth factors from the microenvironment, loss of apoptotic signals and the recruitment of endothelial progenitor cells [15].

**Figure 1:**
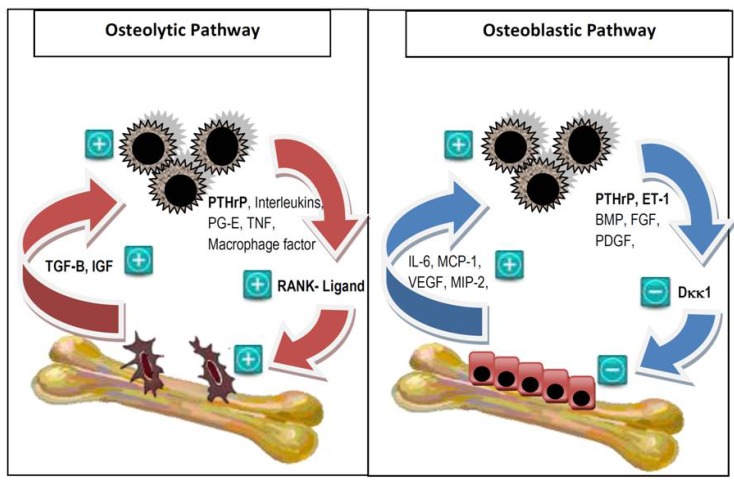
The vicious cycle of bone metastases. In the osteolytic vicious cycle, tumor cells secrete parathyroid hormone-related peptide (PTHrP) and other factors including interleukins, prostaglandin E, tumor necrosis factor and macrophage-stimulating factor. PTHrP induces osteoclastogenesis by upregulation of RANK-L. The activated osteoclasts in turn produce TGF-β and IGF, which promotes cancer cell growth. In the osteoblastic vicious cycle, breast cancer cells produce osteoblast-stimulating factors such as bone morphogenic protein (BMP), fibroblast growth factor (FGF) and platelet-derived growth factor (PDGF). PTHrP is also overexpressed. It activates ET-1, which down regulates Dkk1, a negative regulator of osteoblastogenesis. The activated osteoblasts in turn produce factors including interleukin-6 (IL-6), monocyte chemotactic protein-1 (MCP-1), vascular endothelial growth factor (VEGF), macrophage inflammatory protein-2 (MIP-2); which facilitate breast cancer cell colonization and survival upon arrival in the bone microenvironment. In reality, there is a complex interplay between the two cycles [11,19,20,21]. Reproduced with permission from the *Journal of Breast Cancer: Targets and Therapy* [18].

Osteolytic and osteoblastic metastases result in excessive bone resorption and formation respectively, both at the expense of quality bone formation, mineralization and organization. Radiologically, approximately 48% of bone metastases from breast cancer are purely osteolytic, 13% are purely osteoblastic and 38% are mixed osteoblastic and osteolytic [16]. Histologically and biochemically the two processes coexist irrespective of lytic or blastic radiological appearance [17]. Key mediators of the osteolytic tumor pathway include the parathyroid hormone-related peptide (PTHrP), which upregulates RANK-L from osteoblasts and stromal cells, resulting in down regulation of osteoprotegerin, activation of osteoclasts, production of TGF-β and IGF which in turn promotes tumor cell growth and further release of PTHrP, creating a “vicious cycle of bone metastases” [18] (Figure 1). The osteoblastic pathway is less well studied and much of its research has been focused on prostate cancer. Several mediators produced by tumors are thought to play important roles in the pathogenesis, including endothelin-1 (ET-1), bone morphogenic protein (BMP), fibroblast growth factor (FGF) and PDGF, as well as the Wnt protein pathway through negative regulation of Dickkopf-1 (Dkk1) [19].

An improved understanding of the pathophysiology of bone metastases from breast cancer has ushered the development and clinical use of bone-targeted agents in this area and the specific pathways elucidated provide potential targets for future bone-targeted therapy. 

## 3. Treatment of Bone Metastases from Breast Cancer

### 3.1. Integration of Local and Systemic Therapy

The optimal treatment of bone metastases from breast cancer involves the integration of local, systemic anti-cancer therapy, bone-targeted agents and supportive care through a multidisciplinary team of surgeons, radiation oncologists, medical oncologists, palliative care physicians, radiologists, cancer nurses and coordinators. Treatment is palliative and is aimed at preventing SREs, reducing pain and suffering, preventing disability and improving quality of life.

### 3.2. Prevention of Skeletal-Related Events

#### 3.2.1. Bisphosphonates

Bisphosphonates are potent osteoclast inhibitors and an important class of bone-targeted agents used to reduce the frequency of SREs, improve bone pain and serve as an established treatment for hypercalcemia of malignancy [22,23] (Table 1). Non-nitrogen containing bisphosphonates, such as clodronate and etidronate, are converted intracellularly into methylene-containing analogs of adenosine triphosphate (ATP), which accumulate within macrophages and osteoclasts causing direct apoptosis [18]. Nitrogen-containing bisphosphonates, including pamidronate, ibandronate and zoledronic acid, also inhibit farnesyl diphosphate synthase, a rate-limiting enzyme of the mevalonate pathway, preventing protein prenylation of small guanosine triphosphatase (GTPase) such as Ras, Rho and Rab, which are important signaling proteins that regulate cell survival in osteoclasts [18,24]. *In vitro*, at higher concentrations, nitrogen-containing bisphosphonates inhibit osteoblasts, epithelial and endothelial cells as well as breast tumor cells, in part explaining their potential anti-tumor properties [24].

**Table 1:** Bisphosphonates are defined by their P-C-P conformation, which renders them high affinity to the hydroxyapatite in the bone mineral. Bisphosphonates contain two side chains, R1 being the variable structure that determines the potency of the compound (top left of each structure), and R2 being the short addition that increases the bone affinity (bottom left of each structure). Nitrogen-containing R1 improves the potency by at least 100 fold, and OH-containing R2 significantly increases the affinity to bone. Additional abbreviations: MBC, metastatic breast cancer; IV, intravenous; PO, oral. Reproduced with permission from the *Journal of Breast Cancer: Targets and Therapy* [18].

Class	Simple bisphosphonate		Nitrogen-containing bisphosphonate (N-BP)			
Structure	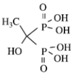	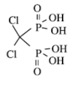	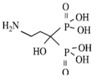	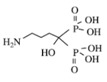	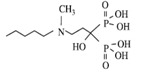	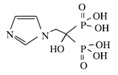
Generic Name	Etidronate	Clodronate	Pamidronate	Alendronate	Ibandronate	Zoledronic acid
Product Name	Didronel^®^	Bonefos^®^	Aredia^®^	Fosamax^®^	Bondronat^®^	Zometa^®^
Relative Potency	1	10	100	1000	10,000	100,000
Possible dosing in MBC	Not indicated	PO 1600–3200 mg daily in single/divided dose	IV 90 mg once every 3–4 weeks	Not indicated	PO 50 mg daily IV 6 mg monthly	IV 4 mg once every 3–4 weeks

**Figure 2:**
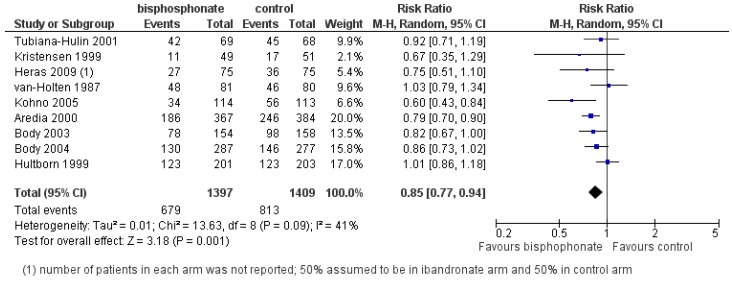
Forest plot of comparison: Overall risk of SREs (excluding hypercalcemia) from breast cancer bone metastases: bisphosphonate *versus* control. Reproduced with permission from the ^©^Cochrane Collaboration [25].

The effects of bisphosphonates on SREs have been extensively studied in metastatic breast cancer over the last two decades using a variety of agents. In a 2012 Cochrane systematic review and meta-analysis, data from 19 randomized controlled trials (RCTs) and 6646 patients were incorporated to evaluate the effects of bisphosphonates or denosumab on SREs from breast cancer bone metastases [25] (Figure 2). For women with advanced breast cancer and clinically evident bone metastases, bisphosphonates significantly reduced the incidence and rate of SREs (excluding hypercalcemia) by 15% as compared to placebo control (risk ratio (RR) 0.85; 95% CI 0.77–0.94; *p* = 0.001) [25]. Efficacy in reducing SREs was demonstrated for both parenteral (RR 0.83; *p* = 0.008) and oral (RR 0.84; *p* = 0.0007) routes of administration compared to control. Individual drug effects on SREs were shown for intravenous (IV) zoledronic acid 4 mg (RR 0.59), IV pamidronate 90 mg (RR 0.77), IV ibandronate 6 mg (RR 0.80), oral ibandronate (RR 0.86) and oral clodronate (RR 0.85) [25]. Few trials have directly compared agents.

A large multi-center randomized, double-blind, placebo-controlled trial of patients with bone metastases from breast cancer and multiple myeloma (*n* = 1130) led by Rosen *et al.* [26] compared 4 mg or 8 mg IV zoledronic acid to 90 mg IV pamidronate, every 3–4 weeks for up to two years. After a protocol modification due to concerns about renal toxicity with the 8 mg zoledronic acid, 4 mg zoledronic acid was shown to be equivalent in efficacy in terms of SREs and tolerability including incidence of renal impairment, when compared to pamidronate in the overall population [26]. In the lytic metastases from breast cancer subgroup (*n* = 528), zoledronic acid produced a significant prolongation of time to first skeletal related event (SRE) (310 *versus* 174 days; *p* = 0.013), significant reduction in skeletal morbidity rate (1.2 *versus* 2.4 events; *p* = 0.008) and a significant reduction in the SRE rate (*p* = 0.010) when compared to pamidronate [27]. Skeletal morbidity rate was significantly lower when zoledronic acid was combined with radiotherapy (0.47 *versus* 0.71 events, *p* = 0.018) or with hormone therapy (0.33 *versus* 0.58 events, *p* = 0.015), suggesting synergism between zoledronic acid and other anti-cancer therapies in preventing skeletal complications [26]. In a more recent phase III trial, the zoledronic acid *versus* oral ibandronate comparative evaluation (ZICE) study (*n* = 1405), oral ibandronate was shown to be inferior to zoledronic acid in terms of the primary endpoint of SRE rate (0.543 *versus* 0.444, HR (hazard ratio) 1.22; 95% CI 1.04–1.45; *p* = 0.017) [28].

The question of when to start a bisphosphonate, and when to stop have, yet to be answered by RCTs. In the exploratory retrospective analysis of the zoledronic acid *versus* pamidronate trial led by Rosen *et al.* [26], patients with one prior SRE were found to be at significantly higher risk (HR 2.08) of developing an on-study SRE than patients with no prior SRE [29]. This suggests starting bisphosphonates early may be warranted rather than waiting for a SRE to occur [18]. The American Society of Clinical Oncology (ASCO) guidelines, the Cancer Australia National Breast and Ovarian Cancer Centre (NBOCC) guidelines and the International Expert Panel guidelines all recommend starting bisphosphonates at the first radiographic sign of bone metastasis [30,31,32] (Table 2). As for the duration of bisphosphonates, there is currently a paucity of data on their use beyond 2 years, which is the treatment duration most commonly set in RCTs. However, this should not be a contraindication to continual therapy in individual patients and is encouraged by consensus guidelines [30,31,32]. The standard dosing of zoledronic acid is 4 mg every 3–4 weeks [30]. A recent randomized controlled trial (RCT) from Italy demonstrated non-inferiority of reduced frequency dosing at every 12 weeks in the second year [33].

In the 2012 Cochrane meta-analysis, few serious adverse events were reported and many were disease or chemotherapy related. Fever and asymptomatic hypocalcemia were the most commonly reported side-effects in women receiving IV pamidronate. Gastrointestinal toxicity was the most frequently reported side-effect of oral bisphosphonates, while acute-phase reactions were more common with IV bisphosphonates [25]. When calcium and vitamin D supplementation were not given, hypocalcemia was more common with zoledronic acid (39% *versus* 7%) compared to placebo [34], however no significant hypocalcemia was seen when calcium and vitamin D supplementation was instituted [27]. Renal toxicity was the main issue with IV zoledronic acid with incidence at 8.5% [35] and was related to dose and infusion time [26]. Osteonecrosis of the jaw (ONJ) was rare at 1.4% after objective assessment [35]. Established guidelines recommend cessation of bisphosphonates prior to invasive dental treatments or avoidance of such procedures during bisphosphonate therapy [36]. 

**Table 2:** Existing guideline recommendations for bisphosphonate use in metastatic breast cancer patients with bone metastases. Additional abbreviations: CT, computed tomography; MR, magnetic resonance; ZOL, zoledronic acid; IBA, ibandronate; PAM, pamidronate; CLO, clodronate; DMB, denosumab. Reproduced with permission from the *Journal of Breast Cancer: Targets and Therapy* [18].

	When to start?	Which bisphosphonate?	When to stop?
ASCO Guidelines 2011 [30]	Breast cancer + radiographic evidence of bone destruction: Lytic disease on X-ray Abnormal bone scan with CT/MR showing bone destruction	IV PAM 90 mg every 3–4 weeks OR IV ZOL 4 mg every 3–4 weeks OR SC DMB 120 mg every 4 weeks	Until evidence of substantial decline in patient’s general performance status
International Expert Panel Guidelines 2008 [32]	Breast cancer + first sign of radiographic evidence of bone metastases, even if patient is asymptomatic	Nitrogen-Bisphosphonate IV preferable (ZOL, IBA, PAM) PO for patients who cannot or need not attend hospital care (CLO, IBA)	Continue beyond 2 years but always based on individual risk assessment; should not discontinue treatment once SRE occurs

While bisphosphonates as a group significantly reduced incidence and rates of SREs, they do not affect survival in women with bone metastases from breast cancer (RR 1.01; 95% CI 0.92–1.11) [25].

#### 3.2.2. Denosumab

Denosumab, a fully human monoclonal antibody to RANK-L, has been shown in preclinical studies and clinical trials to inhibit osteoclast-mediated bone destruction [37]. 

**Figure 3:**
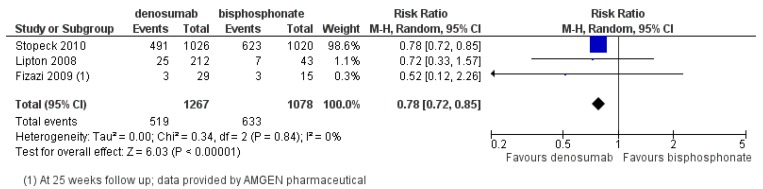
Forest plot of comparison: Overall risk of SREs in breast cancer bone metastases: denosumab *versus* bisphosphonate. Reproduced with permission from ^©^Cochrane Collaboration [25].

Its superior suppression of bone turnover (urinary N-telopeptide <50 nM) compared to zoledronic acid (71% *versus* 29%) was demonstrated in a randomized Phase II trial involving breast cancer, prostate cancer and multiple myeloma patients [38]. In a landmark Phase III trial led by Stopeck *et al.* [35], 2046 patients with bone metastases from breast cancer were randomized to subcutaneous (SC) denosumab 120 mg or IV zoledronic acid 4 mg every 4 weeks. Denosumab significantly delayed first on-study SREs compared to zoledronic acid and the study met both its primary endpoint of non-inferiority (HR 0.82, 95% CI 0.71–0.95; *p* < 0.001) and secondary endpoint of superiority (HR 0.82, *p* = 0.01). Denosumab extended the median time to development of first on-study SRE compared to zoledronic acid (32.4 *versus* 26.4 months) [35]. In addition, denosumab prolonged the time to developing moderate or severe pain compared to zoledronic acid (HR 0.78; *p* = 0.002) [39]. The 2012 Cochrane meta-analysis included 3 RCTs and 2345 patients comparing denosumab and IV bisphosphonates, showed a significant reduction in the risk of developing a SRE by 22% favoring denosumab (RR 0.78; 95% CI 0.72–0.85; *p* < 0.00001) [25] (Figure 3). 

The incidence of adverse events were similar between denosumab and zoledronic acid in the study by Stopeck *et al.* [35]. There was no significant difference in the rate of ONJ (2% *versus* 1.4%, *p* = 0.39) and denosumab was associated with significantly less renal toxicity (4.9% *versus* 8.5%, *p* = 0.001) and fewer acute-phase reactions (10.4% *versus* 27.3%) [35]. Toothache and hypocalcemia were more common with denosumab and for the latter, adequate daily calcium and vitamin supplementation was emphasized. The most common adverse reactions in patients receiving denosumab were fatigue, asthenia, hypophosphatemia and nausea [35]. 

Denosumab’s improved efficacy over zoledronic acid, ease of administration and more favorable renal toxicity profile have resulted in the ASCO guidelines recommending it as a first-line option in the management of bone metastases from breast cancer [30].

Key points for clinical practice:

- Zoledronic acid is the most potent and effective bisphosphonate in preventing SREs. Standard dose is given IV 4 mg every 3–4 weeks for 2 years and to continue if performance status remains adequate;
- Denosumab given 120 mg SC every 4 weeks, has superior efficacy over zoledronic acid in preventing SREs;
- Calcium and vitamin D supplementation could prevent treatment related hypocalcemia;
- While ONJ is rare at 2% or less, invasive dental procedures should be avoided during bisphosphonate or denosumab therapy;
- Bisphosphonates do not improve survival in women with metastatic breast cancer.

### 3.3. Bone Pain and Quality of Life

Intractable bone pain occurs in 50%–90% of patients suffering from bone metastases from breast cancer [40]. Bone pain can be poorly localized, of deep aching quality and patients often experience episodes of stabbing discomfort particularly worse at night not necessarily relieved by lying down [40]. The unique pathophysiology of bone pain involves spinal cord astrocytosis, enhanced neuronal activity through c-Fos expression and sensitization of the central dorsal horn of the spinal cord mediated by dynorphin, a pro-hyperalgesic peptide [41]. It is believed that both tumor-induced damage as well as tumor-produced factors such as endothelin-1 have important roles in the pathophysiology of bone pain [21]. 

#### 3.3.1. Bisphosphonates and Denosumab

A 2002 Cochrane meta-analysis specifically examining bisphosphonate effects on bone pain evaluated the effects of etidronate, pamidronate and clodronate in 30 RCTs encompassing 3682 patients with breast, prostate and lung cancers, multiple myeloma and cancer of unknown primary [42]. There was significant pain relief among patients with metastatic breast cancer who received bisphosphonate therapy (OR (odds ratio) 1.83, 95% CI 1.11–3.04). In the subgroup analysis of the three bisphosphonates, the response was significant for oral clodronate (OR 3.26, 95% CI 1.80–5.09), but not for intravenous pamidronate (OR 2.35, 95% CI 0.77–7.15) and the trend was unfavorable for etidronate (OR 0.28, 95% CI 0.01–7.67) [42]. However, Lipton *et al.* demonstrated in two RCTs (*n* = 751) that pamidronate significantly reduced the pain score (−0.07, *p* = 0.015) and the analgesia score (−0.06, *p* = 0.001) at 24 months [43]. Zoledronic acid has been shown to provide significant and sustained pain relief, and improve quality of life [44]. In addition, it demonstrated efficacy as a second-line agent after failure on pamidronate or clodronate [45]. Both oral and intravenous ibandronate were shown to reduce bone pain, peaking within 8–12 weeks and providing the longest time of sustained pain relief for at least 96 weeks [46]. Quality of life was significantly improved in patients who received intravenous ibandronate [47]. In the study by Stopeck *et al.*, while denosumab delayed the onset of pain compared to zoledronic acid, the median time to pain improvement was similar between treatment arms (82 *versus* 85 days: HR 1.02; *p* = 0.72) [35].

#### 3.3.2. External Beam Radiotherapy

External beam radiotherapy is an established treatment for bone pain secondary to bone metastases, with pain relief commonly achieved within 4–6 weeks. Re-treatment is possible if pain recurs [48]. A single fraction (8 Gy/1 fraction) was shown in a systematic review to be equivalent to multiple fractions (20 Gy/5 fractions) in achieving an overall response in pain (58% *versus* 59%, 95% CI 0.95–1.03), but the retreatment rate was 2.5-fold higher after single fraction treatment (*p* < 0.00001) [49]. A meta-analysis established 8 Gy as the standard dose in a single fraction after demonstrating its superior pain response rates compared to 4 Gy [50]. Re-treatment with single fraction radiotherapy was recently demonstrated to be non-inferior to multiple fractions [51]. Given its equal efficacy, patient convenience and cost effectiveness, single fraction radiotherapy using a dose of 8 Gy to provide palliation from painful bone metastases is supported by the Guidelines from the American Society of Radiation Oncology [52]. 

#### 3.3.3. Radiopharmaceuticals

Bone-targeted radiopharmaceuticals, such as strontium-89, samarium-153 and radium-223 have been developed for palliation of refractory bone pain. These are thought to act as a substitute for hydroxyapatite in bone, with greater uptake in osteoblastic metastases where new reactive bone is formed [18]. While the clinical evidence is most established in metastatic prostate cancer, one study involving 100 patients (40 with metastatic breast cancer) randomized to strontium-89 or samarium-153 showed improvement in performance status (Karnofsky score +20) and reduction in pain (visual analog scale −4) with more favorable results for osteoblastic than mixed metastases [53]. 

### 3.4. Spinal Cord Compression

Spinal cord compression is a potentially devastating complication which occurs in up to 8% of patients with metastatic breast cancer [54]. A multidisciplinary team approach with experienced surgeons, radiation oncologists, medical oncologists, palliative care physicians, cancer nurses and coordinators is often necessary. Optimal management consists of high dose corticosteroids, magnetic resonance imaging to confirm diagnosis, prompt surgical decompression and radiotherapy [18]. In a landmark RCT led by Patchell *et al.* [55], surgery followed by radiotherapy demonstrated a significantly better post-treatment ambulatory rate (84% *versus* 57%, *p* < 0.001) compared with radiotherapy alone, with significantly higher continence rate (OR 0.47, *p* = 0.016), superior functional ability (OR 0.24, *p* = 0.0006) and superior motor strength (OR 0.28, *p* = 0.001) [55]. Survival time was also significantly longer in the combined modality group (126 days *versus* 100 days, OR 0.60, *p* = 0.033) [55]. However, patients with very radiosensitive tumors, multiple areas of spinal cord compression or total paraplegia for longer than 48 h were excluded from the study. Therefore, combined modality treatment with upfront surgery should be offered for fit and functional patients with spinal cord compression, while radiotherapy alone is best reserved for the unfit, already incapacitated patients with poor prognosis [18].

### 3.5. Systemic Endocrine and Chemotherapy

Effective systemic anti-cancer therapy is paramount in the management of bone metastases. While chemotherapy is an integral part of systemic treatment, the role of endocrine therapy is particularly important in bone-only or bone-predominant metastases from breast cancer [18]. Among patients with recurrent breast cancer, those with estrogen receptor (ER)-positive tumors are twice as likely to develop bone metastases as those with ER-negative tumors [56]. Microarray studies in breast cancer also showed that bone metastases occur far more frequently in ER-positive tumors (luminal type 68%), compared with human epidermal growth factor receptor 2 (HER2) positive tumors (20%), basal tumors (7%) and normal molecular subtypes (6%) [57]. The genes upregulated for ER-positive bone metastases are entirely different from those for HER2-positive or basal subtype bone metastases, suggesting a distinct molecular pathway for ER-positive tumors to metastasize to bone [18,57]. Current guidelines recommend endocrine therapy in preference to chemotherapy for women with ER-positive advanced breast cancer, except in the presence of rapidly progressive visceral disease [58]. In the Breast Cancer Trials of Oral Everolimus-2 (BOLERO-2) study published in 2012, the inhibition of the mammalian target of rapamycin (mTOR) pathway using everolimus in addition to exemestane has been demonstrated as an effective systemic treatment for patients with ER-positive metastatic breast cancer who were previously endocrine resistant [59]. Further research in the molecular pathways of bone metastases from ER-positive tumors, including the phosphatidylinositol 3-kinase (PI3K) and mTOR pathways, may provide insights to new therapeutic targets to bone metastases.

### 3.6. Novel Agents and Future Directions

Approximately 55% of breast cancers exhibit TGF-β activity via a 153-gene TGF-β response signature [18]. The overproduction of this multi-function cytokine in the setting of bone metastases from breast cancer induces osteolysis and angiogenesis via mothers against decapentaplegic homolog 3 (Smad 3), which in turn drives epithelial-mesenchymal transition and tumor invasion via multiple cell signaling pathways [60]. TGF-β monoclonal antibodies or tyrosine kinase inhibitors (TKI) strongly inhibit bone metastases from basal like breast cancer in mouse models [61]. These are a promising new class of agents that may help halt the “osteolytic vicious cycle” and are currently entering into early phase clinical trials [18,62]. 

Src is a non-receptor tyrosine kinase that promotes cellular proliferation, differentiation, motility and survival. High levels of Src are implicated in breast cancer osteoclastic activity and activation of endothelial growth factor receptor, HER2, PI3K/mTOR pathways [18]. Src activation was also demonstrated to be associated with late-onset bone metastases in breast cancer [63]. Dasatinib, a multi-targeted Src TKI, had been shown *in vivo* to inhibit osteoclast differentiation and rapidly lowers calcium levels [64]. Several phase I and II trials of dasatinib are currently running, evaluating its role as a bone-targeted agent in addition to zoledronic acid in the treatment of bone metastases from breast cancer [62]. Saracatinib is a dual specific Src/abl TKI which is also being studied in several phase II trials in bone metastases from breast cancer, evaluating its role as a bone-targeted agent compared to zoledronic acid, and as systemic anti-cancer therapy in addition to aromatase inhibitors [18,62]. 

The Wnt pathway plays an important role in osteoblastogenesis. The production of a key protein of the pathway, Dkk1, was first shown to be associated with lytic bone lesions from patients with multiple myeloma [65]. Dkk1 secretion by breast cancer cell lines and high circulating levels were subsequently shown be associated with osteolytic metastases from breast cancer [66]. A clinical trial of a Dkk1 neutralizing antibody BHQ880 is ongoing in patients with lytic lesions from multiple myeloma (NCT00741377) [62]. Further studies are required to evaluate this pathway as a potential therapeutic target in the treatment of bone metastases from breast cancer.

Other potential novel agents include Cathepsin K inhibitors and CXCR4 antagonists, both showing good preclinical effects on bone turnovers and entering early phase clinical trials [18,65]. New biomarker assays may help guide clinicians to select high risk patients for bone metastasis complications and allow better use of bone-targeted agents, refining the use of “personalized medicine” [18].

Key points for clinical practice:

- Bisphosphonates and denosumab improve pain in women with bone metastases from breast cancer;
- Bisphosphonates may improve quality of life, as was demonstrated with IV ibandronate;
- 8 Gy single fraction external beam radiotherapy is an effective means of palliation for bone pain;
- Combined surgery and radiotherapy for spinal cord compression is superior to radiotherapy alone in terms of functional outcomes;
- Optimal treatment of bone metastases involves integration of bone-targeted agents with local and systemic therapy and supportive care through a multidisciplinary team.

## 4. Prevention of Bone Metastases in Advanced Breast Cancer without Skeletal Involvement

The 2012 Cochrane meta-analysis included three studies which evaluated oral bisphosphonates in women with advanced breast cancer without clinically evident bone metastasis [25]. The three RCTs comprised of 320 evaluable patients who received oral clodronate or oral pamidronate compared to placebo. The pooled meta-analysis showed no significant reduction in the incidence of skeletal metastases (RR 0.99; 95% CI 0.67 to 1.47; *p* = 0.97) and no significant difference in survival (RR 0.91; 95% CI 0.75 to 1.11; *p* = 0.36). One study assessed quality of life using a validated questionnaire and found no significant difference between oral pamidronate and placebo control [67]. 

Given the available evidence, the Cancer Australia NBOCC Guidelines do not support the use of bisphosphonates to prevent bone metastases or SREs in women with metastatic breast cancer without clinically evident bone metastasis [31]. Whether more potent modern agents such as zoledronic acid or denosumab can be effective in this setting has not been formally tested in RCTs.

Key points for clinical practice:

- Current evidence do not support the use of bisphosphonates to prevent bone metastases in women with advanced breast cancer without bone metastasis.

## 5. Prevention of Bone Metastases in Early Breast Cancer

### 5.1. Preclinical and Translational Evidence

Dormant cancer cells are an important source of local and systemic breast cancer recurrence [68,69]. The bone marrow provides a unique microenvironment and acts as a niche or sanctuary for disseminated tumor cells (DTC) through a complex interplay of bone and tumor-derived growth factors and cytokines [70]. In an individual patient data pooled analysis of 4703 patients, bone marrow micrometastasis at the time of early breast cancer diagnosis was shown to correlate with increased risk of disease recurrence and poor prognosis [71]. 

Preclinical studies have suggested that bisphosphonates may hinder the development of bone metastases by a direct anti-tumor effect and by modifying the bone microenvironment to become a less accommodating host to cancer cell survival and proliferation [72,73]. There is evidence *in vitro* that bisphosphonates can inhibit tumor adhesion, invasion, induce tumor apoptosis and exert an anti-angiogenic effect [72]. Bisphosphonates may also have immunomodulatory effects, with continual activation of gamma-delta effect or T cells after a single dose of zoledronic acid in an *ex vivo* model of disease-free breast cancer patients [74]. Zoledronic acid, the nitrogen-containing bisphosphonate, demonstrated synergy with chemotherapy when it caused a 10-fold increase in tumor apoptosis *in-vitro* when administered 24 h after doxorubicin [75].

Translational studies also demonstrated the effects of zoledronic acid in reducing the prevalence and survival of DTCs in the bone marrow [76]. In a phase II RCT involving 120 women with stage II or III breast cancer, the addition of zoledronic acid to neoadjuvant chemotherapy was associated with a higher rate of elimination of DTCs from the bone marrow (70% *versus* 53%, *p* = 0.054) [77]. In another study, zoledronic acid after adjuvant chemotherapy significantly reduced the prevalence of DTCs at 12 and 24 months compared to baseline (*p* < 0.001) [78].

These preclinical and translational evidence collectively provided rationale for conducting clinical trials examining bisphosphonates in the adjuvant setting, to prevent metastases and ultimately improve overall survival. 

### 5.2. Adjuvant Bisphosphonate Trials

#### 5.2.1. Oral Clodronate and Ibandronate

Three RCTs commenced in the 1990s examining the effects of clodronate 1600 mg daily as adjuvant therapy for early breast cancer produced discordant results. In the study by Diel *et al.*, where 302 early breast cancer patients with detectable tumor cells in the bone marrow were randomized to 2 years of oral clodronate or placebo, oral clodronate initially improved bone metastasis-free survival (*p* = 0.003) [79] which became statistically insignificant after 8.5 years of follow-up (HR 0.90, *p* = 0.770) [80]. It did however produce a durable improvement in overall survival (OS) (HR 0.50, *p* = 0.04) [79,80]. The Powles *et al.* study, randomized 1069 patients to 2 years of oral clodronate or placebo, oral clodronate produced significant improvements in both bone metastasis-free survival (HR 0.692, *p* = 0.043) and OS (HR 0.743, *p* = 0.041) [81,82]. However, in the Saarto *et al.* study, where 299 patients were randomized to 3 years of oral clodronate and placebo, clodronate did not improve metastasis-free survival (HR 1.23, *p* = 0.35) and OS (HR 1.33, *p* = 0.13), yet was associated with a significant increase in visceral metastasis (HR 1.61, *p* = 0.015) [83,84]. This study was criticized for its methodology and imbalance of baseline characteristics between treatment arms, with the clodronate group having more ER-negative patients (35% *versus* 25%), more post-menopausal women (52% *versus* 43%) who did not receive chemotherapy in this study [18]. In a meta-analysis published in 2007, adjuvant clodronate did not significantly improve bone metastasis-free survival (HR 0.68, 95% CI 0.38–1.23) or OS (HR 0.75, 95% CI 0.31–1.82) although the trend was favorable [85]. 

The National Surgical Adjuvant Breast and Bowel Project (NSABP) B-34 was the largest RCT conducted using adjuvant clodronate, where 3323 women were randomized to 3 years of oral clodronate 1600 mg daily or placebo. The results published in 2012 after a median follow-up of 90.7 months showed no difference in disease-free survival (DFS) (HR 0.91, *p* = 0.27) or OS (0.84, *p* = 0.13) [86]. However, in a pre-planned subgroup analysis of women aged 50 years or older, the clodronate arm showed significantly reduced bone metastasis-free interval (HR 0.62, *p* = 0.027), non-bone metastasis-free interval (HR 0.63, *p* = 0.014) but not OS (HR 0.80, *p* = 0.094). The study investigators hypothesized that adjuvant clodronate may have anti-cancer benefits for older postmenopausal women with a low estrogen bone microenvironment [86].

In the German Adjuvant Intergroup Node-Positive (GAIN) Study, 3023 patients with node-positive breast cancer receiving adjuvant dose-dense chemotherapy were randomly assigned 2:1 to adjuvant oral ibandronate 50 mg daily for two years or observation [87]. There were no significant differences in DFS (HR 0.945, *p* = 0.589) or OS (HR 1.040, *p* = 0.803) between the two arms. However, a trend towards improved DFS was observed in postmenopausal women older than 60 years (HR = 0.75, 95% CI 0.49–1.14) or women younger than 40 years (HR = 0.70, 95% CI 0.44–1.13), some of whom were given ovarian suppression therapy with a luteinizing hormone-releasing hormone (LHRH) agonist, rendering a low estrogen bone microenvironment [87]. 

#### 5.2.2. Zoledronic Acid

The Austrian Breast and Colorectal Cancer Study Group trial-12 (ABCSG-12) was the first RCT to report the effects of adjuvant zoledronic acid in early breast cancer. 1803 premenopausal women with ER-positive early breast cancer treated with either tamoxifen or anastrozole in combination with ovarian suppression using the LHRH agonist goserelin were randomized to receive 3 years of intravenous zoledronic acid 4 mg every 6 months or observation [88]. Gnant *et al.* showed that after 76 months of median follow-up, women who received zoledronic acid had a significantly improved DFS (HR 0.73, *p* = 0.021) and OS (HR 0.59; *p* = 0.042) compared to endocrine therapy alone [89,90]. A pre-planned subgroup analysis showed that the survival benefit of zoledronic acid was restricted to women older than 40 years at study entry (*n* = 1390) and no benefit was seen in women under the age of 40 years (*n* = 413) [89,90]. Gnant hypothesized that very young women may have incomplete ovarian suppression hence a higher estrogen bone microenvironment, which may explain the ineffectiveness of zoledronic acid in this group [90,91]. 

Three companion trials, the Zometa-Femara Adjuvant Synergy Trials (Z-FAST, ZO-FAST and E-ZO-FAST) conducted across North America, the UK, Europe and worldwide evaluated the effects of immediate *versus* delayed zoledronic acid 4 mg every 6 months for 5 years in postmenopausal women with early breast cancer receiving an aromatase inhibitor. The smaller Z-FAST (*n* = 602) and E-ZO-FAST (*n* = 527) studies achieved their primary endpoint of increased bone mineral density but did not show a significant difference in DFS with immediate zoledronic acid [92,93]. However, the larger ZO-FAST (*n* = 1065) study demonstrated a significant reduction of DFS events (HR 0.66, *p* = 0.0375) with immediate zoledronic acid, and fewer local and distant recurrences compared to delayed zoledronic acid which was initiated after a fracture or reduced bone mineral density [94].

The Adjuvant Zoledronic Acid to Reduce Recurrence (AZURE) trial led by Coleman *et al.* [95] was the largest RCT conducted using adjuvant zoledronic acid. 3360 patients with early breast cancer were randomized to standard adjuvant systemic therapy with or without zoledronic acid, given 4 mg every 3 to 4 weeks for 6 doses then every 3 to 6 months to complete 5 years. The addition of zoledronic acid did not significantly improve DFS (HR 0.98; *p* = 0.79) or OS (HR 0.85; *p* = 0.07) in the overall population [95]. However, pre-planned subgroup analysis showed that in women who were at least 5 years postmenopausal (*n* = 1041), zoledronic acid significantly improved DFS (HR 0.75; *p* = 0.02) and OS (HR 0.74; *p* = 0.04) [95]. 

While the AZURE and NSABP-B34 adjuvant bisphosphonates trials were demonstrably negative in the overall population, benefit in the postmenopausal or ovarian suppression subgroup is consistently observed across multiple trials. This raises the hypothesis that the greatest anti-tumor benefit of adjuvant bisphosphonates is in a low estrogen microenvironment. A low estrogen state may, through unknown mechanisms, allow alterations in bone microenvironment, “the soil”, to become less conducive to tumor “seeding” and metastases [70,90].

#### 5.2.3. Meta-Analyses

The 2012 Cochrane meta-analysis including 9 RCTs did not show any significant benefit of adjuvant bisphosphonates in preventing bone metastases (RR 0.94; *p* = 0.36), or overall disease recurrence (RR 0.97; *p* = 0.75) or OS (RR 0.84; *p* = 0.11) in the overall early breast cancer population [25]. Two recent meta-analyses of RCTs of adjuvant zoledronic acid have yielded conflicting results. Yan *et al.* [96] meta-analyzed 5 studies (*n* = 7354) and demonstrated no significant improvement in OS, DFS or bone metastasis-free survival with the use of adjuvant zoledronic acid compared to control. However, in the postmenopausal subgroup, the addition of zoledronic acid significantly improved DFS (RR 0.763, *p* < 0.001), locoregional recurrence (RR 0.508; *p* = 0.001) and distant recurrence (HR 0.744; *p* = 0.003), but there was no significant improvement in OS (RR 0.811; *p* = 0.286) [96]. Valachis *et al.* [97] meta-analyzed 15 studies (*n* = 9197) and demonstrated a significant improved OS (HR 0.81; *p* = 0.007) but no difference in DFS (HR 0.86; *p* = 0.15) or incidence of bone metastases (HR 0.94; *p* = 0.74) [97]. The differences in inclusion criteria may have accounted for the discrepancy of results in the two meta-analyses. Of note, the high levels of heterogeneity of studies (I^2^ = 67.3% for DFS in Yan *et al.*, I^2^ = 55% for DFS in Valachis *et al.*) [96,97] is an issue for both meta-analyses, and has made it difficult for a firm conclusion to be drawn. Nevertheless, meta-analyses have concluded that adjuvant zoledronic acid is well tolerated in the adjuvant setting, with the rate of ONJ at 0.52% [97], which is lower than in the metastatic setting [35]. 

Overall, the meta-analyses conducted based on published data do not provide definitive answers to the question and the available evidence do not support the routine use of adjuvant bisphosphonates in unselected patients with early breast cancer.

### 5.3. Future Directions

#### 5.3.1. Individual Patient Data Meta-Analysis

While the results of other adjuvant bisphosphonate studies such as the Southwest Oncology Group-0307 (SWOG-0307) and the German Simultaenous Study of Gemcitabine-Docetaxel Combination Adjuvant Treatment, as well as Extended Bisphosphonate and Surveillance (SUCCESS) trials are awaited, an individual patient data meta-analysis will be required to definitively answer the question on adjuvant bisphosphonates in early breast cancer. This is necessary in order to minimize publication bias and the effects of heterogeneity of included studies, as were shown to be problematic in the meta-analyses conducted using published data. Particular attention is required in the subgroup of women with a low estrogen bone microenvironment, either through natural menopause or medical ovarian suppression. 

#### 5.3.2. Denosumab

Denosumab has been shown to improve bone mineral density and is safe and well tolerated when compared to placebo in patients with non-metastatic breast cancer [98]. The effects of adjuvant denosumab on recurrence and survival are currently being investigated, including the large RCT, the Study of Denosumab as Adjuvant Treatment for Women with High Risk Early Breast Cancer Receiving Neoadjuvant or Adjuvant Therapy (D-CARE), with results expected after 2016 [62]. 

#### 5.3.3. Biomarkers

Genomic and proteomic profiling have been shown to have prognostic potential correlating with disease progression as well as predictive potential for specific treatment benefits [99]. In the future, these technologies may help guide clinicians to select patients to treat aggressively with bone-targeted agents and to selectively omit unnecessary treatment. While health care costs in oncology are rising globally, personalized medicine in the molecular age may potentially be cost-effective and cost-saving [100]. 

Key points for clinical practice:

- Current evidence do not support the routine use of adjuvant bisphosphonates in unselected women with early breast cancer;
- The incidence of ONJ in the adjuvant setting is very rare, in the order of 0.52%;
- Adjuvant bisphosphonates may provide survival benefit in the subgroup of women with low estrogen bone microenvironment, either through natural menopause or ovarian suppression;
- Individual patient data meta-analysis will be required to definitively address this question.

## 6. Conclusions

Bone metastasis from breast cancer is a common condition and is associated with incurable disease, significant complications, morbidity and reduced quality of life. Treatment of bone metastases is palliative and is aimed at reducing SREs, preserving mobility and improving quality of life. Greater understanding of the pathophysiology of bone metastases has led to the discovery and clinical utility of safe and effective bone-targeted agents such as bisphosphonates and denosumab. The integration of bone-targeted agents with other local, systemic anti-cancer therapy and supportive care is important for the optimal treatment of bone metastases. While potentially more effective bone-targeted agents are being developed, prognostic and predictive biomarkers may help guide future directions on personalized treatment of bone metastases from breast cancer. 

While there is no evidence for the use of bisphosphonates for the prevention of bone metastases in advanced breast cancer without skeletal involvement, its use in the adjuvant setting has generated worldwide interest in recent years. As preclinical and translational data suggested potential therapeutic effects of bisphosphonates on tumor cells and the bone microenvironment, multiple large scale RCTs involving bisphosphonates as adjuvant therapy in early breast cancer have been completed. While bisphosphonates have failed to conclusively show any survival benefit in unselected early breast cancer patients, consistent benefits in reducing bone recurrence and/or improving survival have been observed in the subgroup of women with a low estrogen bone microenvironment, either through natural menopause or ovarian suppression. As we await the results of a few more ongoing RCTs, an individual patient data meta-analysis will be most useful to answer this important question in early breast cancer management and bone metastasis prevention. 
